# Humanoid Cognitive Robots That Learn by Imitating: Implications for Consciousness Studies

**DOI:** 10.3389/frobt.2018.00001

**Published:** 2018-01-26

**Authors:** James A. Reggia, Garrett E. Katz, Gregory P. Davis

**Affiliations:** ^1^Department of Computer Science, University of Maryland, College Park, MD, United States; ^2^Maryland Institute for Advanced Computer Studies (UMIACS), University of Maryland, College Park, MD, United States

**Keywords:** machine consciousness, artificial consciousness, neural network gating mechanisms, cognitive robots, cognitive phenomenology, imitation learning, computational explanatory gap, working memory

## Abstract

While the concept of a conscious machine is intriguing, producing such a machine remains controversial and challenging. Here, we describe how our work on creating a humanoid cognitive robot that learns to perform tasks *via* imitation learning relates to this issue. Our discussion is divided into three parts. First, we summarize our previous framework for advancing the understanding of the nature of phenomenal consciousness. This framework is based on identifying computational correlates of consciousness. Second, we describe a cognitive robotic system that we recently developed that learns to perform tasks by imitating human-provided demonstrations. This humanoid robot uses cause–effect reasoning to infer a demonstrator’s intentions in performing a task, rather than just imitating the observed actions verbatim. In particular, its cognitive components center on top-down control of a working memory that retains the explanatory interpretations that the robot constructs during learning. Finally, we describe our ongoing work that is focused on converting our robot’s imitation learning cognitive system into purely neurocomputational form, including both its low-level cognitive neuromotor components, its use of working memory, and its causal reasoning mechanisms. Based on our initial results, we argue that the top-down cognitive control of working memory, and in particular its gating mechanisms, is an important potential computational correlate of consciousness in humanoid robots. We conclude that developing high-level neurocognitive control systems for cognitive robots and using them to search for computational correlates of consciousness provides an important approach to advancing our understanding of consciousness, and that it provides a credible and achievable route to ultimately developing a phenomenally conscious machine.

## Introduction

In this paper, we use the word “consciousness” to mean specifically phenomenal consciousness unless explicitly indicated otherwise. The term “phenomenal consciousness” has been used historically to refer to the subjective qualities of sensory phenomena, emotions, and mental imagery, for example the color of a lemon or the pain associated with a toothache (Block, 1995). Searle has presented a list of essential/defining features of consciousness, including subjectivity, unity, qualitativeness, situatedness, and sense of self (Searle, 2004), and a detailed analysis of this term can be found in Chapter 3 in Tani (2017). Recent work in philosophy has argued for an extended view of phenomenology that includes one’s cognitive processes and hence is referred to as cognitive phenomenology, as we will elaborate below. In the following, we focus on conscious qualities specific to cognitive phenomenology in particular, as opposed to the more historically emphasized aspects of consciousness such as sensory qualia.

How can research based on cognitive humanoid robots contribute to our understanding of consciousness? Consciousness is not well understood at present, and many philosophers have questioned whether computational studies or cognitive robots can play a significant role in understanding it. Such arguments cannot be refuted at present because there is currently no convincing implementation of instantiated consciousness in a machine, as described in Reggia (2013). Conversely, none of these past arguments appear sufficiently strong to convince many current investigators that machine consciousness is impossible (Reggia et al., 2015). For this reason, it seems prudent to us to push ahead investigating this issue until the matter can be definitively resolved one way or the other, and it is in that context that we describe our research efforts below.

Here, we describe how our past and ongoing work on creating a humanoid cognitive robot that learns to perform tasks *via* imitation learning relates to consciousness studies. Our key contribution here is to expand and develop a concrete framework for investigating the nature of consciousness in cognitive robots. Our discussion is divided into three parts. First, we summarize our framework for advancing the understanding of the nature of phenomenal consciousness based on studying the computational explanatory gap (CEG) (Reggia et al., 2014). The main goal in this work is to identify neurocomputational correlates of consciousness. We believe that identifying such correlates will be possible in cognitive robots, based on concepts that have emerged recently in the philosophical field of cognitive phenomenology, and we explain why that is so.

The core idea of our framework for studying consciousness in robots is that investigating how high-level cognitive processes are implemented *via* neural computations is likely to lead to the discovery of new computational correlates of consciousness. Accordingly, in the second part of this paper, we describe a cognitive robotic system that we recently developed that learns to perform tasks by imitating human-provided demonstrations. This humanoid robot uses cause–effect reasoning to infer a demonstrator’s goals in performing a task, rather than just imitating the observed actions verbatim. Its cognitive components center on top-down control of a working memory that retains the explanatory interpretations that the robot constructs during learning. Because, as we explain below, both cause–effect reasoning and working memory are widely recognized to be important aspects of conscious human thought, we suggest that exploring how the cognitive and memory mechanisms embodied in our imitation learning robot provide an excellent test of our framework for studying consciousness in machines.

Finally, in the third part of this paper, we describe our recent and ongoing work that is focused on converting our robot’s imitation learning cognitive system into purely neurocomputational form, including its causal reasoning mechanisms and cognitive control of working memory. We summarize our initial results exploring the feasibility of this idea. Based on these results, we argue that the top-down cognitive control of working memory, and specifically its gating mechanisms, is potentially an important computational correlate of consciousness in humanoid robots that merits much further study. We conclude that developing neurocognitive control systems for cognitive robots and using them to search for computational correlates of consciousness provides an important approach to advancing our understanding of consciousness, and that it provides a credible and achievable route to ultimately developing a phenomenally conscious machine.

## A Computational Approach to Understanding the Nature of Consciousness

In the following, we propose a *computational* framework for investigating consciousness. We begin by summarizing the concept of a CEG, and we explain why recent advances by philosophers interested in cognitive phenomenology makes this barrier relevant to consciousness studies. We then describe our proposed framework for studying consciousness that is based on identifying its computational correlates.

### Computation, Mind, Brain, and Body

We have previously suggested that there is an important obstacle to understanding the prospects for machine consciousness that we call the CEG (Reggia et al., 2014). The CEG is defined as our current inability to understand how higher-level cognitive computations supported by the brain can be accounted for by lower-level neurocomputational processes. We use the term “higher-level cognition” to refer to cognitive processes including decision-making, reasoning, intent-directed problem solving, executive control of working memory contents, plan generation, and language. These cognitive processes are viewed by many psychologists as being consciously accessible. In contrast, we use the term “lower-level neurocomputational processes” to refer to the types of computations that can be implemented using artificial neural networks like those currently studied in fields such as neuroscience, computer science, psychology, and engineering.

The CEG is related to past work in philosophy, neuroscience, and psychology, addressing various aspects of the mind–brain problem. In philosophy, the CEG differs from the *philosophical explanatory gap*, the latter referring to the difficulty we have in explaining how physical systems in the objective world can support the subjective qualities of consciousness (Levine, 1983). The philosophical explanatory gap relates to how difficult it is to understand how subjectivity can emerge from the brain or potentially from other physical systems such as machines. The CEG differs in that it is *not* a mind–brain issue. Instead, the CEG is our current inability to understand how *computations* supporting high-level cognitive processes like those described above can be implemented *via* the lower-level *computations* that neural networks provide. Put otherwise, it deals only with computational issues, and it applies both to people and to machines. Historically, philosophers have tended to deprecate the CEG, characterizing it as part of the “easy” problem of interpreting how the brain generates intelligent behavior (Chalmers, 1996). This viewpoint fails to account for why the CEG has been so difficult to bridge over the last 50 years in spite of an enormous research effort to do so. It also ignores the possibility that the philosophical explanatory gap and the CEG are not two independent issues, but that instead, the CEG might ultimately prove relevant to understanding the mind–brain problem. It is this latter issue that we discuss in the following, arguing that the CEG is relevant to obtaining a deeper understanding of the mind–brain problem. More recently in philosophy, work in *cognitive phenomenology* has argued that our phenomenal experiences are not limited to classical qualia such as those of sensory perception, but also include high-level cognition (Bayne and Montague, 2011; Jorba and Vincente, 2014; Chudnoff, 2015). It is this idea more than anything else that makes the CEG, a purely computational issue, of relevance to understanding consciousness. Accepting that some facets of cognition reach conscious awareness is what makes computational studies of the CEG important in consciousness studies. The *hypothesis* guiding our work described below is thus that bridging the CEG provides a pathway to deeper comprehension of consciousness and eventually possibly even a phenomenally conscious machine. This hypothesis makes research that is directed at creating neurocomputational implementations of higher-level cognitive processes, including our own work with adaptive cognitive robots as described below, relevant to the issue of phenomenal consciousness.

The CEG also relates to recent work in the neurosciences and psychology. In the neurosciences, our current state of knowledge can be characterized as knowing a lot about how high-level cognitive functions correlate with different macroscopic brain areas (e.g., language comprehension and Wernicke’s area, planning and prefrontal cortex) and a great deal about the microscopic neurobiological networks in these same areas. However, what we do not currently understand is how the brain implements the high-level cognitive processes using the underlying neural circuitry. We view this situation as an example of the CEG, quite separate from any considerations about consciousness. In psychology, related work has been done to investigate the differences between information processing that is unconscious and information processing that is conscious (Dehaene and Naccache, 2001; Baars, 2002). Unconscious information processing is fast and can support multiple concurrent tasks, and these tasks can be done simultaneously without interfering with each other. It tends to involve localized brain regions and is often not reportable (people cannot explain how they carried out a task). In contrast, conscious information processing is much slower, restricted to one task at a time, involves widespread cortex activation, and is generally taken to be cognition that a subject can report. Again, we view such findings as being related to the CEG. The computational properties associated with unconscious processes often match up well with those of neural computations (e.g., the opaqueness or “non-reportability” of what a neural network has learned). The computational properties during conscious, reportable cognitive activities are much closer to what is seen with symbolic artificial intelligence (AI) systems, and do not relate well to how neural networks process information. To be clear, we are not suggesting that consciousness can be explained by symbolic reasoning or language—we just intend to convey that conscious, reportable cognitive activities need to be accounted for by resolving the CEG. Further, we are only considering the existence of consciousness in adults and do not relate our work to the mechanisms underlying the emergence of consciousness in infants.

Symbolic AI models are often used on computers devoid of any remotely human- or animal-like embodiment. However, all compelling and widely accepted examples of consciousness in the real world occur in embodied biological systems. Even proponents of cognitive phenomenology still consider it plausible that conscious cognitive processing has some basis in sensorimotor experience (Prinz, 2011). From a purely practical standpoint, studying the CEG in the context of embodied robotic systems may be the most efficient route to ecologically valid input data for cognitive models. And it stands to reason that humanoid robots in particular will be best for studying machine consciousness that is as human-like as possible. At a deeper level, there are serious philosophical positions that consider embodiment to be intrinsically related to cognitive phenomenology (Nagataki and Hirose, 2007). In sum, studying cognition in the context of humanoid robots specifically may be an important factor in bridging the CEG and potentially understanding/engineering consciousness.

### A Framework for Investigating Consciousness

An implication of the ideas presented in the preceding section is that much recent research involving neurocomputational models of high-level cognition becomes relevant to comprehending the properties of consciousness. The basic idea is that these computational investigations could discover neurocomputational mechanisms occurring with phenomenally conscious aspects of cognition that are not also found to be present during cognitive processes that are unconscious. We have proposed elsewhere that this could provide examples of computational correlates of consciousness, in the same way that neuroscientists have identified neural correlates of consciousness (Reggia et al., 2014, 2016).

A *computational correlate of consciousness* has been defined previously to be an aspect of information processing associated with conscious but not unconscious information processing (Cleeremans, 2005). In general, a computational correlate of consciousness is not the same thing as a neural correlate as described by neuroscientists. Previously described neural correlates have included biological concepts that are not computational, e.g., regions of the brain, biochemical processes, and electrical activity patterns in the brain (Chalmers, 2000). On the other hand, the definition of computational correlates above is fairly general. For example, it might include logical reasoning algorithms like those studied in traditional AI. In this context, previous researchers have suggested that cognitive processes can be separated into neurocomputational processes representing unconscious facets of cognition, and symbolic processes representing conscious facets of cognition (Kitamura et al., 2000; Sun, 2002; Chella, 2007), i.e., symbolic information processing is viewed as a computational correlate of consciousness. However, from our perspective, such models do not provide a way to bridge the CEG. The central idea in bridging the CEG as we defined it above is to identify how higher-level reasoning is implemented *via* underlying, purely neurocomputational mechanisms, much as the brain does. This is the crux of the matter.

Thus, in the rest of this paper we use the term “computational correlates of consciousness” to refer solely to neurocomputational mechanisms that occur only with conscious facets of higher-level cognitive processes and are *not* found with neurocomputational processes involved with other unconscious information processing (not with neurocomputational mechanisms associated with implementing the normal pupil light reflex, for example). These correlates may be implemented in the brain, but are independent of the physical mechanisms that implement them (robot control circuitry, biological brain circuitry, and so forth). Our proposal is that uncovering computational correlates of consciousness will provide insight into the nature of consciousness (as per cognitive phenomenology) and possibly even the development of a plausibly conscious physical machine.

We have recently given a fairly detailed description of previously proposed computational correlates of consciousness (Reggia et al., 2016) and refer the interested reader to that work. Here, we just briefly give a few examples that illustrate the central ideas involved. One widely known proposal is that *global information processing* is a computational correlate of consciousness, inspired by findings that information processing during conscious mental activities (and not unconscious cognitive processes) occurs widely across the cerebral cortex and is also correlated with enhanced communication between brain regions (Baars et al., 2003; Massimini et al., 2005; Tagliazucchi et al., 2016). Another prominent past suggestion is that *information integration* in a neural network is what distinguishes conscious from unconscious systems in general (Tononi, 2004). Still others have suggested that having a *self-model* is a computational correlate (Searle, 2004; Samsonovich and Nadel, 2005), even showing that physical robots controlled by neural networks can pass the “mirror test” of self-awareness used with animals (Takeno, 2013). Other researchers have suggested that higher-order representations of one’s knowledge about the world correlate with consciousness (Cleeremans et al., 2007; Pasquali et al., 2010). Additional studies have argued that *attention mechanisms* are potential computational correlates (Taylor, 2007; Haikonen, 2012). All of these ideas are intriguing and may provide important clues as to the fundamental nature of consciousness, and the fact that so many ideas are emerging in this area is quite encouraging.

## A Cognitive Humanoid Robot that Learns by Imitating

In the previous section, we described a framework for studying aspects of consciousness based on developing computational/robotic systems that account for high-level cognitive functions in neurocomputational terms. To pursue this approach, two things are needed: a physical robotic system that supports some aspects of high-level cognitive functionality, and an underlying neural control mechanism that implements that functionality.

Here, we describe our recent work on the first of these two requirements: Our efforts to create a cognitive humanoid robot that that can be used to explore consciousness-related and other issues (Katz et al., 2017a,b). Why would one want to consider studying the CEG in a robot instead of simply going the easier route of computer simulations? One answer is that a cognitive system in a robot is embodied: It interacts with and causally acts on a real external environment, and in that sense there is a true “mind-body” problem, at least to the extent that one is willing to call a robot’s cognitive control system a mind. Further, it has been claimed that the ability to ground a cognitive robotic system’s symbols in the robot’s sensory data stream is a computational correlate of consciousness (Kuipers, 2008). While this suggestion is controversial (Chella and Gaglio, 2012), it suggests that some computational correlates may be particularly evident in a cognitive system that interacts with the real world as part of a physical system.

Our own robot learns to perform tasks by imitating human-provided demonstrations. During learning, it uses cause–effect reasoning to infer a demonstrator’s goals in performing a task, rather than just imitating the observed actions literally. Importantly for our own research as described in subsequent sections, the robot’s cognitive components center on top-down control of a working memory that retains the explanatory interpretations that the robot constructs during learning. We first briefly summarize this work here and then, in the next section, we relate this work to the search for computational correlates of consciousness.

### Imitation Learning *via* Cause–Effect Reasoning

Our work in robotics is motivated in part by the fact that it is currently very hard to program humanoid robots to carry out multi-step tasks unless one has a great deal of expertise in robotics. A potential solution to this problem is to use imitation learning (learning from demonstrations) rather than manually programming a robot. With imitation learning, a robot watches a person perform the task to be learned, and then imitates what it observed. An important mode of imitation learning occurs at the sensorimotor level, when the learning robot closely imitates the motions, gestures, and perhaps even the facial expressions of the demonstrator. Much work on robotic imitation learning has focused on this level. While important, this level does not involve an understanding of the demonstrator’s intentions, and hence suffers from limited ability to generalize to new situations where the robot must use different actions to carry out the same intentions.

Figuring out what a demonstrator’s goals are is a kind of cause–effect reasoning known as “abduction” in AI. The issue is to postulate what the demonstrator’s goals are in a way that is consistent with these goals *causing* the observed actions. AI researchers have extensively studied cause–effect reasoning (also called abductive reasoning) like this, including its use to infer the goals of an acting agent (Kautz and Allen, 1986; Peng and Reggia, 1990; Carberry, 2001). While some aspects of cognition have been simulated during past studies of imitation learning (Chella et al., 2006; Friesen and Rao, 2010; Dindo et al., 2011), to our knowledge, the utility of causal reasoning during imitation/goal learning has not been studied substantially. However, in other application domains such as medical diagnosis or circuit fault localization, causal reasoning systems often rely on finite databases of background knowledge that exhaustively describe all of the possible causal events that might occur. In robotic imitation learning, this amounts to a finite list of general purpose primitive actions that a demonstrator or robot might perform, as well as the direct causal relationships between those actions and higher-level goals, and the possible objects that might be present in the environment. The full spectrum of possible goals, actions, and objects involved in general human imitation learning is probably too rich and variable to be adequately encoded in a finite database. However, for specific applications, there will likely be a finite set of possible objects to be manipulated and a finite set of actions and goals that can be applied to those objects. In this case, it is feasible to adapt existing causal reasoning approaches to robotic imitation learning. Moreover, individual actions and goals within a finite list can still admit continuous-valued parameters, such as object positions and rotations, in order to approximate some of the richness and variability inherent in true human imitation learning. This is the causal knowledge representation supported in our existing work described below. A detailed description of the encoded knowledge as well as the algorithms used in our applications can be found in Katz et al. (2017a). Future work on underlying neural mechanisms for the causal reasoning functionality could incorporate generative neural models to produce novel situation-specific actions that need not be anticipated in a finite database by a human knowledge engineer.

**Figure 1:**
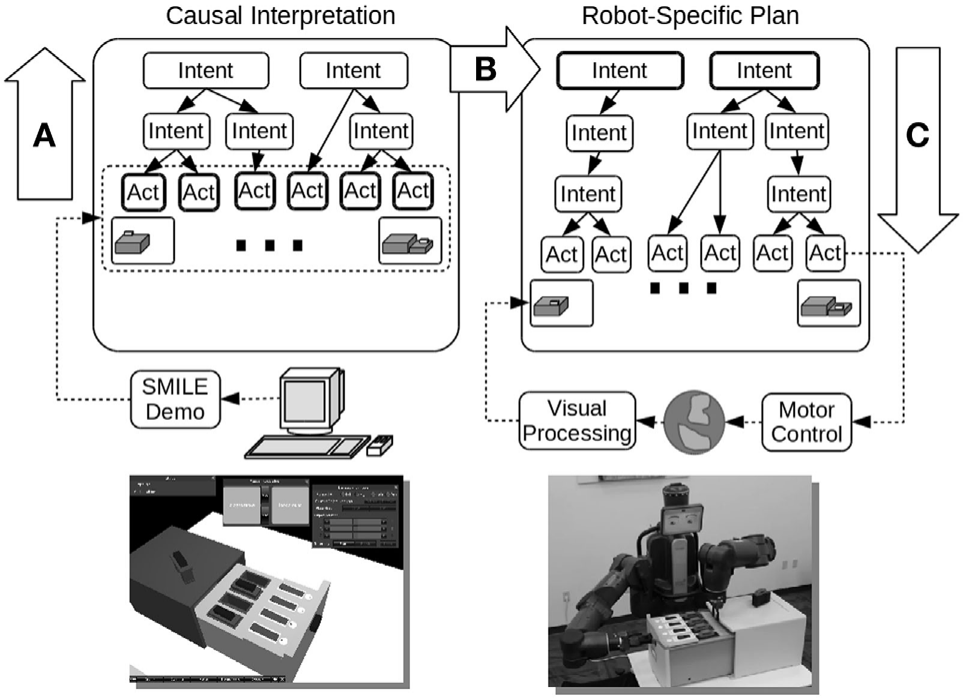
A top-level view of CERIL, the cognitive portion of our imitation learning robotic system. The abductive reasoning processes (infer the causes from the effects) are shown on the left: they produce a hierarchical causal network that represents at the top an *explanation* for the observed demonstrator’s actions. After learning, this explanation can be used to guide plan generation in related but modified situations, as illustrated on the right. Figure from Katz et al. (2017a).

In this context, we recently suggested that causal reasoning is an important part of cognitively oriented imitation learning. To examine whether this idea can support imitation learning, we developed and studied an approach to imitation learning based on abductive cause–effect reasoning as illustrated in Figure 1 (Katz et al., 2016, 2017a). During the observation of a demonstration, our approach assembles a parsimonious explanation for what was observed where the demonstrator’s intentions (goals) serve to explain the actions performed by the demonstrator. We refer to our cognitive learning model as CERIL, for Cause–Effect Reasoning in Imitation Learning. The basic idea with CERIL is that the inferred demonstrator’s goals (rather than the specific actions the demonstrator performed) can subsequently be used in related but new situations that may need different specific action sequences to achieve the same goals. Given that our primary interest here is in the role played by high-level cognition during imitation learning, our focus is on that and we largely take low-level sensorimotor processing as a given.

Figure 1 illustrates an example of CERIL learning about and then subsequently performing actions on a disk drive docking station. CERIL learns to maintain this disk drive dock, for example replacing hard drives that experience a hardware fault. The objective of learning is to replicate a teacher’s *goals* in subsequent post-learning situations rather than to produce a literal repetition of the demonstrator’s actions. For example, if the demonstrator replaces a failing disk drive, CERIL must do the same thing, even if the spare drive has to come from a different location, and even if the faulty drive is in a different slot. CERIL may use a different arm for certain steps, or transfer objects from one “hand” to another, even though the demonstrator did not take these specific actions.

As illustrated at the bottom left in Figure 1, a person provides a demonstration to CERIL by using a graphical computer program with GUI controls in which the demonstrator manipulates objects on a virtual tabletop (Huang et al., 2015a,b). CERIL uses the event record from this demonstration to infer an explanation for the demonstrator’s actions in terms of high-level goals for the shown task (labeled A in Figure 1). The high-level goals/intentions/schemas have parameters, such as with *grasp (object, location, gripper)*. In constructing explanations, CERIL uses pre-defined goals/intentions and their sub-goals/sub-intentions that are defined *a priori* in its knowledge base. Explanations typically consist of a novel *sequence* of instantiated/grounded high-level goals that CERIL constructs through abductive causal reasoning. In particular, the inference process is an extended version of parsimonious covering theory (Peng and Reggia, 1990). The term “parsimony” refers to the fact that the simplest explanations are to be preferred, while “covering” refers to the fact that a plausible explanation must be able to cause (cover) the observed demonstrator actions. Adapting parsimonious covering as the basis of imitation learning required substantial extensions to the original theory (Katz et al., 2017a). These extensions included incorporating real-valued variables such as object locations and orientations, integrating causal chaining and temporal constraints, and accounting for spatial transformations related to manipulating objects.

### Does It Work?

The right side of Figure 1 illustrates what happens after imitation learning of a task is complete. CERIL can learn and retain multiple tasks over multiple environments, but here we just consider the single disk drive task described above as an example. After learning, CERIL can be given situations in the real world that are similar to what it was trained with (labeled B in Figure 1). It will then match its parameterized object models to the objects in the physical environment, which grounds its top-level goals in the new situation. It then uses its grounded explanation (a sequence of goals to be achieved in the order specified) to generate a plan for performing the specific task it has been given by using a hierarchical task network (HTN) planner (Ghallab et al., 2004). This is labeled C in Figure 1. From the viewpoint of parsimonious covering theory, this HTN planning process is using CERIL’s cause–effect relations in the opposite direction from what was done during learning (i.e., reasoning now goes from causes to effects rather than the opposite which was done during learning). Unlike during the learning phase, HTN planning now involves using goals and actions that are specific to the robot, not to the human demonstrator.

We have systematically tested CERIL using a humanoid physical robot (Baxter, Rethink Robotics™; pictured at the lower right of Figure 1) on a set of different tasks, and the detailed results can be found in Katz et al. (2017a). These tasks include learning basic maintenance skills on the disk drive station illustrated above, learning maintenance tasks on a pipe-and-valve plumbing configuration, and learning to construct toy block configurations. In addition, we used computer simulations to test CERIL’s ability to interpret correctly action sequences taken from a data set of 5,000 emergency response plans (Blaylock and Allen, 2005). CERIL was able to function effectively and efficiently in all of these situations (Katz et al., 2017a). Most compelling is that CERIL is often able to learn and generalize to modified initial situations (spare disk is in a different initial location, a different indicator light is on, etc.) from a single demonstration, much as a person can do. Further computational simulations comparing different parsimony criteria have investigated the impact of using different criteria for what it is that makes an explanation “parsimonious” (Katz et al., 2017b), and we are currently conducting an experimental study to compare how CERIL’s learning and subsequent imitation compare to what is done by human subjects in the same situations.

**Figure 2:**
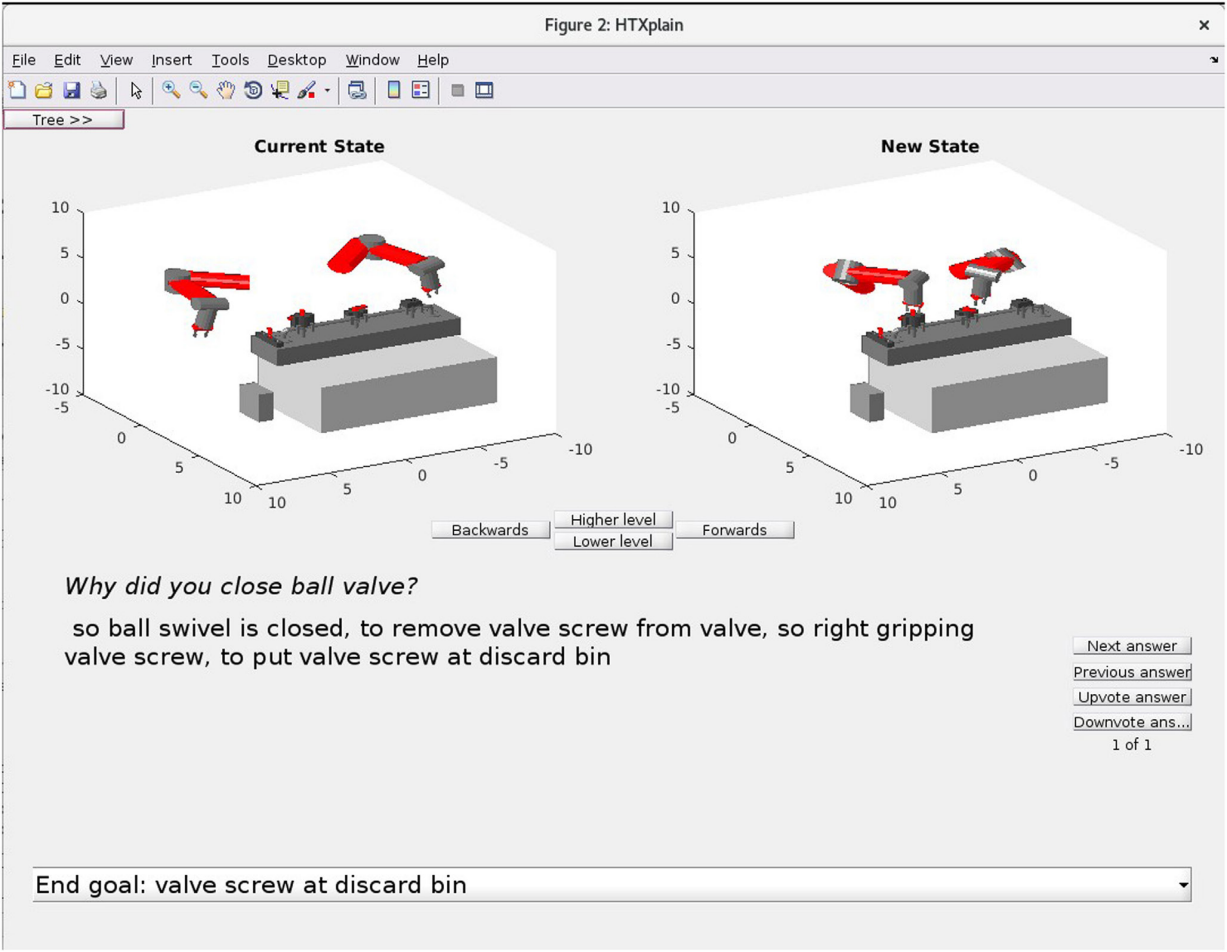
Because of its use of cause–effect knowledge and abductive inference methods that are arguably models of human knowledge and reasoning, CERIL can generate simple intuitive justifications of its actions to a person who is observing a humanoid robot at work. While the English is a bit stilted, in the example shown here CERIL is responding to a question as to why it closed a ball valve by describing its reasons (causative factors based on its goals).

Finally, a potential benefit of using a cognitive model of the kinds of cause–effect reasoning performed by humans during learning and planning is that it should allow a robot to explain to a human observer why it is carrying out certain actions with justifications that are intuitively plausible. Such an ability is critical to making the simulated reasoning mechanisms of robots and other autonomous systems transparent to people, and this transparency is often an important aspect of machine trustworthiness. We have recently introduced methods by which CERIL can justify its actions to a human observer based on “causal plan graphs” (Katz et al., 2017c). Figure 2 gives an example of this action sequence justification ability in its current form for a simple device maintenance task. We believe that such “reportability” of underlying inference processes will ultimately prove to be important to investigating the possibility of machine consciousness. The reason for this is that in experimental psychology, investigators long taken a subject’s being able to report verbally his/her cognitive experiences to be an objective criterion for that subject to be subjectively aware of those experiences (Baars, 1988; Dehaene and Naccache, 2001).

## Bridging the CEG

We believe that the imitation learning humanoid robot described above, when controlled by a purely neurocomputational high-level cognitive control system and lower-level sensorimotor system, provides an excellent context in which to study the CEG and to search for potential computational correlates of consciousness. It uses hierarchical causal knowledge, abductive inference, and intention/goal inference processes, all of which have long been widely viewed as modeling important aspects of human reasoning in general and involved in imitation learning specifically (Kassirer and Gorry, 1978; Peng and Reggia, 1990; Josephson and Josephson, 1994; Meltzoff, 1995; Baldwin and Baird, 2001; Bekkering and Prinz, 2002; Haikonen, 2003; Fuster, 2004; Fogassi et al., 2005; Iacoboni et al., 2005; Walton, 2005; Botvinick, 2008; Katz et al., 2017a). However, the control mechanisms instantiated by CERIL are currently implemented with traditional software: Our robot’s cognitive components are top-down symbolic AI algorithms for abductive inference and plan generation. In order to use our robotic learning system to study the CEG, the existing software needs to be converted into neurocomputational form, something that is currently in progress. At present, we have converted the low-level sensorimotor control of individual robot actions into neural network modules, replacing the corresponding original software with a neural architecture, the DIRECT algorithm, that we have previously studied *via* non-robotic computer simulations (Gentili et al., 2015). Testing of the resulting robotic control system (i.e., the top-down symbolic cognitive components plus the neural sensorimotor components instantiated in our robot) on tasks such as maintenance operations on the disk drive dock and pipe-and-valve system described above show that the robot’s behavior with a neural sensorimotor system is virtually unchanged from the original.

We have concurrently also been studying, so far only *via* non-robotic computer simulations, neural mechanisms for cognitive control of working memory and other behaviors that are intended to serve as purely neurocomputational replacements for CERIL’s existing executive control system. In the rest of this section, we first describe the neurocomputational systems we are developing that are inspired by both cortical and subcortical processes that are believed to underpin human cognitive control mechanisms. We then describe a key hypothesis of our work addressing the CEG: that top-down gating of working memory is an important computational correlate of consciousness. This hypothesis is motivated in part by the recognition by many psychologists that working memory is a significant aspect of conscious human cognition, as we explain further below.

### Neurocomputational Implementation of Top-Down Gating

The current implementation of our robotic system for imitation learning provides a good illustration of the CEG as we portrayed it above: high-level cause–effect reasoning and planning successfully implemented using symbolic AI operations, and low-level sensorimotor control successfully implemented using neural network methods. Given the framework that we have outlined above (see A Framework for Investigating Consciousness), our specific research agenda is clear: search for computational correlates of consciousness by replacing CERIL’s causal reasoning and planning algorithms with a purely neurocomputational system that provides the same functionality. Such a replacement is beyond the reach of current neurocomputational technology and is a very challenging target. However, it provides a concrete example of attempting to bridge the CEG, and in this context it has the potential to reveal candidates for computational correlates of consciousness as per our research framework and cognitive phenomenology.

Given this challenge, we are taking inspiration from what is known about the neurobiological mechanisms underlying human cognitive control. Of course, current understanding of these biological mechanisms is incomplete, but what is known provides a powerful foundation for addressing how CERIL’s mechanisms might be implemented using neural computations. Here, we give two examples of the results we have obtained so far using this approach, explaining for each how they relate specifically to the issue of top-down control of cognitive mechanisms.

**Figure 3:**
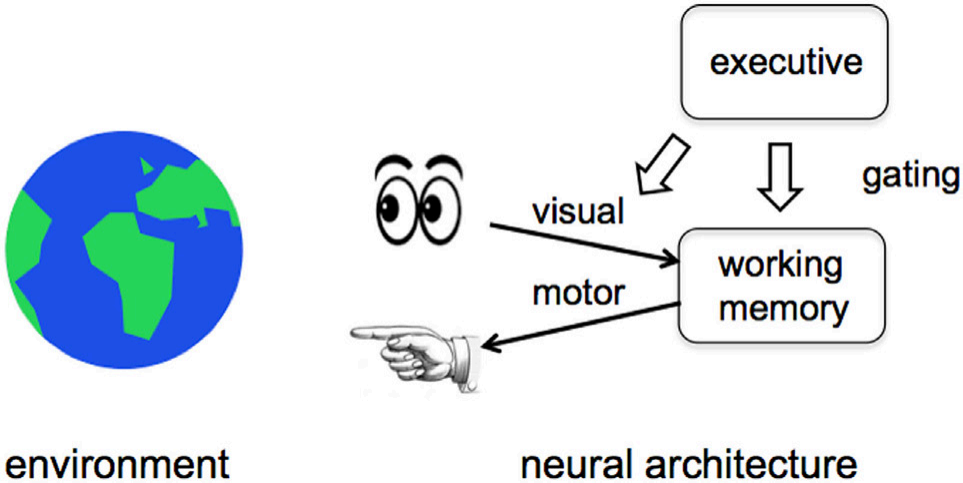
The top-level architecture of GALIS’ neural control system. The operational components of an intelligent agent’s control system, such as visual information processing, motor control, and working memory, are gated by an executive system at the upper right. Our work focuses on the top-down gated control of working memory in particular.

First, we created and studied a neurocomputational system named GALIS that models executive control functions and can be related to the CEG (Sylvester et al., 2013; Sylvester and Reggia, 2016). We have studied this model in computer simulations, and the goal now is to adapt an extended version of the methods used in GALIS as the top-level neural control mechanisms in CERIL. As illustrated in Figure 3, this model is centered on an executive system that gates (turns on or off) the functions of the other components of the system, including working memory. The working memory module is an autoassociative recurrent network that adopts one-step Hebbian synaptic changes to quickly store and recall problem-solving information such as what objects are in the workspace and their locations. The executive control module, of primary interest here, is trained to activate/de-activate the functions of the other components in the system. This gating control mechanism thus determines whether or not inputs are saved in working memory, when information stored in working memory is to be deleted, and when outputs are to be produced. Using Hebbian learning methods, it is possible to “program” GALIS to carry out tasks that require a sequence of motor actions to be executed that are specific to solving a given problem. For example, we trained GALIS to play simple card games that required it to retain in working memory the previous cards that it had seen, and to base decisions about its actions on the contents of working memory. Not only did GALIS perform the task well in solving hundreds of randomly generated card game problems, but it was also found to exhibit some significant similarities to people in terms of how many steps it took to solve card game problems of various difficulty levels (Sylvester and Reggia, 2016) as well as in memory capacity in separate experiments simulating human n-back problem solving (Sylvester et al., 2013).

The executive component, shown at the upper right in Figure 3, is the most interesting aspect of GALIS’ underlying neurocomputational system in the context of the CEG. It exerts top-down control over the functions of other operational parts of the overall system. This executive has an internal structure that is more complex than illustrated in Figure 3. It consists of multiple components, the most important of which is an associative memory that stores task instructions as attractor states. Each instruction indicates which system components should be activated/de-activated (*via* the gating mechanism) at various times during a task in order to solve whatever problem is under consideration. The executive is trained to represent and remember sequences of instructions (“programs”) as sequences of attractor states. Like working memory, learning is based on Hebbian synaptic changes. Subsequently, the executive sequentially visits those learned attractor states in the correct order during problem solving. In effect, this procedural memory allows the executive to learn to represent simple tasks (sequences of instructions or “programs”) as sequences of transient attractor states. This is of special interest in the context of past suggestions that some activity state trajectories in neural systems might be computational correlates of consciousness (Fekete and Edelman, 2011). What our model adds to this suggestion is the specific idea that temporal sequences of *attractors* (itinerant attractor sequences) used by executive modules instantiating top-down gating might be the specific property that makes activity state trajectories become computational correlates of consciousness. This idea is related to recent work suggesting that sequences of attractor states in recurrent neural networks can shed light on controversies surrounding cognitive phenomenology (Aleksander, 2017). The executive system in GALIS is sufficiently robust even in its current implementation to store and use multiple instruction sequences as appropriate as different conditions arise during problem solving.

**Figure 4:**
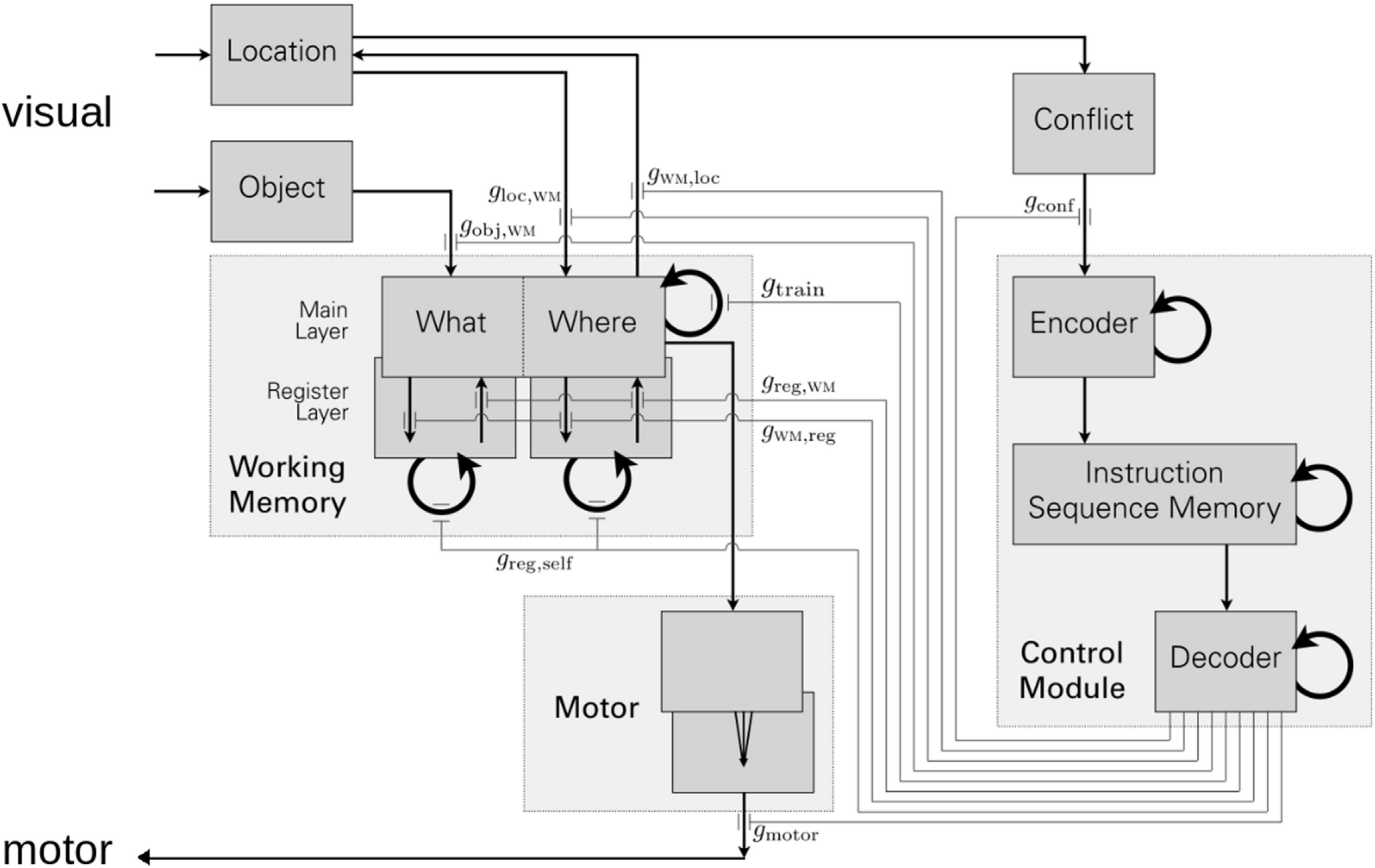
An elaboration of the details of Figure 3 above. Each rectangle with darker gray shading corresponds to a neural module, where thick semi-circular arrows indicate recurrently connected networks. Thin arrows indicate the connectivity between modules. The working memory of Figure 3 is depicted on the left, while the executive system (control module) is shown on the right. The connections leaving the decoder module at the lower right of the illustration implement gating actions as explained in the main text. Figure from Sylvester and Reggia (2016).

Figure 4 elaborates on GALIS’ top-level architecture that is illustrated in Figure 3. Sensory inputs enter at the upper left, and motor control (e.g., “pointing” at a card) leaves at the bottom left. The internal structure of the recurrently connected networks forming working memory is shown, indicating that this memory stores associated pairs of object-location information. The memory for instruction sequences, or “programs,” is a recurrent neural network shown on the right as part of the control module. Not only does it store individual instructions as attractor states (much like the working memory, *via* symmetric synaptic weights produced by one-step Hebbian learning), but it also stores the transitions between one instruction to the next. Representing a sequence of attractor states in memory could be done in various ways, e.g., Tani has suggested that compositionality and discrete action sequences (sequences of a nonlinear neural system’s states) can be supported *via* chaotic dynamics (Tani, 2017). In GALIS, sequencing between instructions is instead based on asymmetric weights on recurrent connections in the instruction sequence memory’s network. These asymmetric weights are learned *via* temporally asymmetric Hebbian learning. Thus, during performance of a task, the instruction memory goes to an attractor state (an instruction) corresponding to a local minimum of the network’s energy function and performs the specific action(s) indicated by that instruction. The underlying energy landscape governing dynamics then shifts, making the current attractor/instruction unstable since it is no longer an energy minimum state. Guided by the learned asymmetric weights, the state of the network then transitions to a new local energy minimum that is the next instruction in the sequence/program. Multiple instruction sequences can be stored simultaneously in GALIS’ control memory. The detailed network structure and equations governing GALIS’ activity dynamics and synaptic changes during learning can be found in Sylvester and Reggia (2016).

Most importantly for our discussion here, as the executive system transitions through an instruction sequence, it exerts top-down influences on the functionality of other modules in the system. This control is exerted by gating connections leaving the executive system and traveling to other parts of the system. These gating connections originate at the lower right in Figure 4 and are labeled *g_x_* in the illustration, where *g_x_* is the activity state of connection *x*. For example, the executive system turns on learning in the working memory, directing working memory to store the currently seen object’s identity and location, by having an output *g*_train_ = 1, while it directs working memory to instead ignore the current visual input by having an output of *g*_train_ = 0. Gating like this in GALIS is implemented *via* “multiplicative modulation” (Akam and Kullmann, 2014), where the *g_x_* values occur in the equations governing activity dynamics and learning in other modules. As an example, if unit *k* in the motor module has an activity *a_k_*, then what the external world actually sees at that time is the value *g*_motor_ × *a_k_* that incorporates *g*_motor_ as a multiplying factor. If *g*_motor_ = 1, then the actual output from unit *k* at that time is *a_k_*, while if *g*_motor_ = 0, the actual output is 0. The specific details of how module functionality is gated in the equations controlling system behaviors are given in Sylvester and Reggia (2016).

The core ideas behind GALIS—using top-down gating patterns to encode instructions, and using itinerant attractors to represent sequences of instructions and other data—make for a highly versatile model of computation that can support symbolic reasoning systems like CERIL. For example, suppose that activity patterns are used to represent individual actions and goals that might occur. Itinerant attractor sequences could then be used to store a list of actions that carry out a particular goal, or the list of goals that might cause a particular action, thereby encoding background causal knowledge. Moreover, during reasoning, a working memory could be used to incrementally accumulate a list of conjectured goals that are mutually consistent and account for all actions observed in a demonstration. Finally, instruction memory could be used to store the sequences of gating patterns that carry out the reasoning algorithms. For example, un-gating learning or activation dynamics could be used to store or retrieve background knowledge, respectively. Similarly, during reasoning, un-gated sequence learning in working memory could be used to append new goals when constructing an explanation, and un-gated interactions between background knowledge, working memory, and conflict detection regions could be used to check for inconsistencies before an explanation is modified. Of course, many more subtleties and details will have to be accounted for in a successful implementation. The foregoing examples are intended just to convey the high-level implementation strategy and bolster our claim to its feasibility.

However, a significant limitation to GALIS’ executive module is its inability to handle ambiguity. There is no need for a complex decision-making process in the card matching task described above because it could be specified with a simple set of deterministic rules to carry out based on the state of the environment. More realistic tasks often necessitate decision-making to resolve conflicts between potential responses, and often depend on reinforcement learning mechanisms to determine the relative value of these responses. For this reason, our second effort to implement neural mechanisms that could replace CERIL’s symbolic algorithms focuses on the role of subcortical structures like the basal ganglia in cognitive control, such as with decision-making and action selection.

Decades of research have implicated the basal ganglia in a wide array of cognitive and motor functions, many of which are associated with conscious processing (Schroll and Hamker, 2013). Most notably, deficits observed in disorders such as Parkinson’s disease suggest a role of the basal ganglia in voluntary movement initiation (Wurtz and Hikosaka, 1986), sequential action performance (Benecke et al., 1987; Jin et al., 2014), attention (Tommasi et al., 2015; Peters et al., 2016), and working memory (Lewis et al., 2005; Gruber et al., 2006). In many cases, the functionality of primitive sensorimotor reflexes in Parkinson’s disease patients is correlated with increases in cognitive impairment, suggesting a decreased ability to exert top-down control over unconscious behavior (Vreeling et al., 1993). In addition, there is evidence that abnormal inhibition in the striatum of the basal ganglia is associated with the conscious compulsions reported in tic disorders such as Tourette’s syndrome (Vinner et al., 2017). Taken together, this evidence suggests that the basal ganglia comprise an important instrument for conscious top-down control over the central nervous system that could offer a number of potential benefits to a neurocognitive system, including a mechanism for biasing attractor landscapes like those used in GALIS toward reward associated trajectories (goal-directed behavior) and controlling the maintenance and capitulation of salient states (working memory). While the neural mechanisms underlying these processes are not fully understood (Goldberg et al., 2013), past computational models incorporating basal ganglia have been shown to capture important behavioral patterns associated with top-down control (Wiecki and Frank, 2013).

We are currently incorporating such a model into GALIS by dividing the executive module into components corresponding to the prefrontal cortex and the basal ganglia. The latter is intended to address the aforementioned ambiguity issues that arise in complex environments by providing a competitive decision-making component that resolves conflicts arising in the former. In addition, such a component functions as a detector of salient states, thereby providing cues for the timing of behavioral execution, serving as a gate on the gating mechanism itself to prevent premature responses or to interrupt ongoing execution when appropriate. This is particularly relevant to the sensorimotor level of imitation learning. As mentioned above, we replaced traditional low-level motion planning with the DIRECT neural algorithm (Bullock et al., 1993; Gentili et al., 2015), which learns in an unsupervised fashion using exploratory “babbling.” Much of the robotic motion planning done during imitation learning of maintenance tasks (like those we described above) requires the use of an inverse kinematics solver that determines a joint trajectory for a given end-effector starting position and target. DIRECT learns this coordinate transformation in a self-organizing map architecture by training on a randomly generated set of joint movements and their consequential end-effector transformations. It computes inverse kinematics by finding a difference vector and adjusting the end-effector position using the transformed kinematic information for the appropriate movements that must be made to reach the goal state. Once trained, the resulting model is capable of producing iterative joint movements that approximate the shortest path to the target position.

We have developed an augmented version of the DIRECT model that controls imitation of coordinated bimanual movements (Gentili et al., 2015) to support end-effector orientations, which are critical to performing the demonstrated tasks. This allows the planner to provide joint trajectories that orient the robot’s grippers for fine motor tasks, such as manipulating screws, coordinating exchanges of objects between grippers, and fitting objects into tight spaces. However, these additional dimensions were found to pose a unique problem due to the rotational limits of the robot’s wrists in the absence of high-level decision-making and top-down control. The DIRECT model is trained to approximate the shortest path to the target position and orientation, but this path may be blocked by the rotational boundary of the wrists, in which case they must be rotated in the opposite direction. Furthermore, a given task may call for a particular rotational direction (for example, unscrewing demands counterclockwise rotation, regardless of the shortest path to the target rotation). Importantly for our work, these considerations motivate the need for top-down control by indicating situations in which top-down control over sensorimotor processing can be used to resolve planning conflicts and override habitual behavior: a gating signal may be used to force the motion planner to take the longer, suboptimal path. It is this kind of context-dependent control over top-down gating that we are currently implementing in the simulated basal ganglia components of our model, which is work in progress.

### Top-Down Gating of Working Memory

A key hypothesis of our work addressing the CEG described above is that the top-down gating of working memory (and potentially of other operational components) is an important computational correlate of consciousness. At the least, we believe that studying this aspect of the CEG will lead to the discovery of such correlates. Why is that?

The term *working memory* can be defined as the memory mechanisms that store and process information for a brief period of time. Human working memory has very strict capacity limitations: Psychologists have found that we can only retain about four separate items in our working memory at any point in time (Cowan et al., 2005). If one tries to store more information, the individual items stored may interfere with each other and, in any case, the items will be replaced or decay away over time as problem solving evolves.

The important point here in terms of our work concerning the CEG is that psychologists consider the information processing done by the working memory system to be part of our *conscious* cognitive processes. They have found that storing, manipulating, and recalling information from working memory is conscious and reportable (Block, 2011; Baddeley, 2012). Thus, according to the tenets of cognitive phenomenology (discussed in Section “Computation, Mind, Brain, and Body”), the computational processes that control working memory deserve consideration as possible computational correlates of consciousness. Further, working memory operations are largely managed *via* cognitive control systems that are biologically most clearly associated with prefrontal cortex “executive functions” that manage other cognitive processes in general (Schneider and Chein, 2003). In terms of the CEG, the issue becomes: can we identify neurocomputational mechanisms that might implement the control of working memory functionality? Elaborating on the hypothesis stated at the beginning of this section, our proposal is that top-down gating like that described above, which determines what is saved and discarded by working memory, furnishes the computational machinery that is used by executive cognitive processes in controlling working memory operations during conscious information processing and is thus a potential computational correlate of consciousness. With top-down gating, an executive module controls the functions of other modules. An executive system may use gating to enable/disable the connectivity between modules, to determine when they remember/forget information, when they generate outputs such as motor actions, and when they learn.

Our specific neurocomputational models described in the preceding subsection envision gating functions, guided by a neurodynamical executive system that sequentially visits attractors that represent instructions (i.e., that represent a procedure for carrying out a task), as corresponding to conscious aspects of cognition that involve working memory. In addition, the gating of working memory in a top-down fashion is reminiscent of the idea of mental causation considered by philosophers deliberating on the topic of free will (Kane, 2005; Murphy et al., 2009). These observations and the finding that control of working memory using top-down gating works effectively in neurocomputational systems and produces behavioral measurement results similar to those observed in humans during n-back memory tasks and card matching tasks (Sylvester et al., 2013; Sylvester and Reggia, 2016) as described above, suggest to us that further investigation of these gating mechanisms may be profitable in the search for computational correlates of consciousness.

## Discussion

Current understanding of phenomenal consciousness is widely recognized to be very incomplete, and its relationship to cognition and the core neuroanatomical structures that support it continue to be the focus of recent work (Spreng et al., 2008; Wang and He, 2014; Gomez-Marin and Mainen, 2016). This holds both with respect to consciousness in people and with respect to issues that surround the question of whether machines or animals can be conscious. The primary suggestion in this paper is that the CEG is an important contributing reason for our limited progress toward a better understanding of phenomenal consciousness. This viewpoint runs counter to some past philosophical arguments that understanding the mechanisms of human cognition will not get us any closer to solving the “hard problem” of consciousness. However, the growing recognition among contemporary philosophers who support the idea of cognitive phenomenology suggests, to us at least, that cognition and consciousness are sufficiently intertwined that computational exploration of the CEG may productively lead to insights about the nature of conscious, both in machines and people. It is for this reason that we have suggested a framework for studying consciousness that is based on searching for neurocomputational correlates of consciousness in cognitive-level machines. Ultimately, this general framework, if applied broadly, may turn out to be critically important to providing new knowledge about our basic notions of consciousness. Our view is that the CEG is a central issue for consciousness studies, and one that merits substantial investigation over coming years. Doing this should lead us to discoveries about the computational correlates of consciousness.

More specifically, in this paper we have emphasized the importance of searching for neurocomputational correlates of consciousness, and suggested that one direction in which such a search may prove to be productive is the investigation of executive gating of working memory functions. To our knowledge, very little past work in cognitive robotics or involving computational modeling has examined this specific issue. There have been past computational studies motivated by higher-order thought (HOT) theory that relate cognitive mechanisms to working memory. But these past neurocomputational models based on HOT theory have, to our knowledge, only developed “metacognitive networks” that *monitor* one another, and have not considered the possibility of top-down gating architectures where executive modules *control* other modules’ actions. Top-down gating as we describe it here also differs from previously proposed computational models of attention, including proposals that the production of an “efference copy” by control mechanisms (Taylor, 2007) or that having multiple components of a system simultaneously focus on a single subject (Haikonen, 2012), are computational correlates of consciousness. Such models do not explicitly focus on using top-down gating as described in this paper as a control mechanism. As we noted earlier, other past related work includes the suggestion that some activity state trajectories in neural systems might be computational correlates of consciousness (Fekete and Edelman, 2011), and the temporal sequences of attractors used by executive modules instantiating top-down gating in our system is consistent with such a suggestion.

There is much room for further work in this area. For example, at the present time the mechanisms by which a cortical/subcortical region may directly or indirectly control/gate the functions of other regions is not completely clear. Gating interactions in the brain could possibly be implemented by direct pathways between cortical areas, indirectly *via* actions of basal ganglia and thalamic nuclei, by functional mechanisms such as synchronized cortical oscillations, or by some mixture of these and other yet-to-be discovered mechanisms. An important future research topic would be to undertake a more detailed examination of the implications of using alternative gating mechanisms. This relates to the broader issue of what features must be incorporated into computational neural network models to make them adequately representative of brain functions. Current neural network technology spans a broad range of biological realism, running from the relatively realistic Hodgkin–Huxley models incorporating spiking neurons with multi-compartment dendritic trees to the relatively implausible use of linear models or backpropagation learning. In our own work, we have tried to strike a balance regarding this issue, but it remains an important question as to the level of complexity and biological realism in neural computation that will ultimately be best related to the investigation of consciousness. Further future work in neuroscience and psychology is also needed to sharpen our understanding of which cognitive processes are conscious and which are not as a prerequisite for validating computational correlates of consciousness.

## Author Contributions

All the authors contributed substantially to this work and agree to be accountable for the content of the work. All the authors reviewed and approved the manuscript.

## Conflict of Interest Statement

The authors declare that the research was conducted in the absence of any commercial or financial relationships that could be construed as a potential conflict of interest.

## References

[B86] Akam T. Kullmann D. (2014). Oscillatory multiplexing of population codes for selective communication in the mammalian brain. Nat. Rev. Neurosci. 15, 111–123. 24434912 10.1038/nrn3668 PMC4724886

[B87] Aleksander I. (2017). “Cognitive phenomenology: a challenge for neuromodelling,” in Proceedings on Aritificial Intelligence and Simulated Behavior, eds Bryson J. De Vos M. Padget K. (Bath, UK), 395–398.

[B1] Baars B. (1988). A Cognitive Theory of Consciousness. Cambridge, UK: Cambridge University Press.

[B2] Baars B. (2002). The conscious access hypothesis. Trends Cogn. Sci. 6, 47–52. 10.1016/S1364-6613(00)01819-2 11849615

[B3] Baars B. Ramsey T. Laureys S. (2003). Brain, conscious experience, and the observing self. Trends Neurosci. 26, 671–675. 10.1016/j.tins.2003.09.015 14624851

[B4] Baddeley A. (2012). Working memory: theories, models and controversies. Annu. Rev. Psychol. 63, 1–29. 10.1146/annurev-psych-120710-100422 21961947

[B5] Baldwin D. Baird J. (2001). Discerning intentions in dynamic human action. Trends Cogn. Sci. 5, 171–178. 10.1016/S1364-6613(00)01615-6 11287271

[B6] Bayne T. Montague M. (eds) (2011). Cognitive Phenomenology. Oxford, UK: Oxford University Press.

[B7] Bekkering H. Prinz W. (2002). “Goal representations in imitation learning,” in Imitation in Animals and Artifacts, eds Dautenhahn K. Nehaniv C. (MIT Press), 555–572.

[B8] Benecke R. Rothwell J. C. Dick J. P. Day B. L. Marsden C. D. (1987). Disturbance of sequential movements in patients with Parkinson’s disease. Brain 110, 361–379. 10.1093/brain/110.2.361 3567527

[B9] Blaylock N. Allen J. (2005). “Generating artificial corpora for plan recognition,” in User Modeling, LNAI, Vol. 3538, eds Ardissono L. Brna P. Mitrovic A. (Edinburgh: Springer), 179–188.

[B10] Block N. (1995). On a confusion about a function of consciousness. Behav. Brain Sci. 18, 227–247. 10.1017/S0140525X00038188

[B11] Block N. (2011). Perceptual consciousness overflows cognitive access. Trends Cogn. Sci. 15, 567–575. 10.1016/j.tics.2011.11.001 22078929

[B12] Botvinick M. (2008). Hierarchical models of behavior and prefrontal function. Trends Cogn. Sci. 12, 201–208. 10.1016/j.tics.2008.02.009 18420448 PMC2957875

[B13] Bullock D. Grossberg S. Guenther F. (1993). A self-organizing neural model of motor equivalent reaching and tool use by a multi-joint arm. J. Cogn. Neurosci. 5, 408–435. 10.1162/jocn.1993.5.4.408 23964916

[B14] Carberry S. (2001). Techniques for plan recognition. User Model. User Adapt. Interact. 11, 31–48. 10.1023/A:1011118925938

[B15] Chalmers D. (1996). The Conscious Mind. Oxford, UK: Oxford University Press.

[B16] Chalmers D. (2000). “What is a neural correlate of consciousness?” in Neural Correlates of Consciousness, ed. Metzinger T. (Cambridge, MA: MIT Press), 17–39.

[B17] Chella A. (2007). “Towards robot conscious perception,” in Artificial Consciousness, eds Chella A. Manzotti R. (Exeter, UK: Imprint Academic), 124–140.

[B18] Chella A. Dindo H. Infantino I. (2006). A cognitive framework for imitation learning. Rob. Auton. Syst. 54, 403–408. 10.1016/j.robot.2006.01.008

[B19] Chella A. Gaglio S. (2012). Synthetic phenomenology and high-dimensional buffer hypothesis. Int. J. Mach. Conscious. 4, 353–365. 10.1142/S1793843012400203

[B20] Chudnoff E. (2015). Cognitive Phenomenology. Routledge Press.

[B21] Cleeremans A. (2005). Computational correlates of consciousness. Prog. Brain Res. 150, 81–98. 10.1016/S0079-6123(05)50007-4 16186017

[B22] Cleeremans A. Timmermans B. Pasquali A. (2007). Consciousness and metarepresentation: a computational sketch. Neural Netw. 20, 1032–1039. 10.1016/j.neunet.2007.09.011 17904799

[B23] Cowan N. Elliott E. Scott Saults J. Morey C. Mattox S. Hismjatullina A. (2005). On the capacity of attention: its estimation and its role in working memory and cognitive aptitudes. Cogn. Psychol. 51, 42–100. 10.1016/j.cogpsych.2004.12.001 16039935 PMC2673732

[B24] Dehaene S. Naccache L. (2001). Towards a cognitive neuroscience of consciousness. Cognition 79, 1–37. 10.1016/S0010-0277(00)00123-2 11164022

[B25] Dindo H. Chella A. La Tona G. Vitali M. Nivel E. Thorisson K. R. (2011). “Learning problem solving skills from demonstration,” in AGI, LNCS, Vol. 6830, eds Schmidhuber J. Thorisson K. R. Looks M. (Berlin, Heidelberg: Springer), 194–203.

[B26] Fekete T. Edelman S. (2011). Towards a computational theory of experience. Conscious. Cogn. 20, 807–827. 10.1016/j.concog.2011.02.010 21388834

[B27] Fogassi L. Ferrari P. F. Gesierich B. Rozzi S. Chersi F. Rizzolatti G. (2005). Parietal lobe: from action organization to intention understanding. Science 308, 662–667. 10.1126/science.1106138 15860620

[B28] Friesen A. Rao R. (2010). “Imitation learning with hierarchical actions,” in Proc. of the 9th Intl. Conf. on Development and Learning (IEEE), 263–268.

[B29] Fuster J. (2004). Upper processing stages of the perception-action cycles. Trends Cogn. Sci. 8, 143–145. 10.1016/j.tics.2004.02.004 15551481

[B30] Gentili R. Oh H. Miller R. Huang D. Katz G. Reggia J. (2015). A neural architecture for performing actual and mentally simulated movements during self-intended and observed bimanual arm reaching movements. Int. J. Soc. Robot. 7, 371–392. 10.1007/s12369-014-0276-5

[B31] Ghallab M. Nau D. Traverso P. (2004). Automated Planning. San Francisco, CA: Elsevier.

[B32] Goldberg J. H. Farries M. A. Fee M. S. (2013). Basal ganglia output to the thalamus: still a paradox. Trends Neurosci. 36, 695–705. 10.1016/j.tins.2013.09.001 24188636 PMC3855885

[B33] Gomez-Marin A. Mainen Z. (2016). Expanding perspectives on cognition in humans, animals and machines. Curr. Opin. Neurobiol. 37, 85–91. 10.1016/j.conb.2016.01.011 26868042

[B34] Gruber A. J. Dayan P. Gutkin B. S. Solla S. A. (2006). Dopamine modulation in the basal ganglia locks the gate to working memory. J. Comput. Neurosci. 20, 153–166. 10.1007/s10827-005-5705-x 16699839

[B35] Haikonen P. (2003). The Cognitive Approach to Conscious Machines. Exeter, UK: Imprint Academic.

[B36] Haikonen P. (2012). Consciousness and Robot Sentience. Singapore: World Scientific.

[B37] Huang D. Katz G. Langsfeld J. D. Oh H. Gentili R. J. Reggia J. (2015a). “An object-centric paradigm for robot programming by demonstration,” in Foundations of Augmented Cognition 2015. LNCS, Vol. 9183, eds Schmorrow D. D. Fidopiastis M. C. (Springer), 745–756.

[B38] Huang D. Katz G. Langsfeld J. D. Gentili R. J. Reggia J. (2015b). “A virtual demonstrator environment for robot imitation learning,” in IEEE Intl. Conf. on Technologies for Practical Robot Applications (TePRA) (IEEE), 1–6.

[B39] Iacoboni M. Molnar-Szakacs I. Gallese V. Buccino G. Mazziotta J. C. Rizzolatti G. (2005). Grasping the intentions of others with one’s own mirror neuron system. PLoS Biol. 3:e79 10.1371/journal.pbio.0030079 15736981 PMC1044835

[B40] Jin X. Tecuapetla F. Costa R. M. (2014). Basal ganglia subcircuits distinctively encode the parsing and concatenation of action sequences. Nat. Neurosci. 17, 423–430. 10.1038/nn.3632 24464039 PMC3955116

[B41] Jorba M. Vincente A. (2014). Cognitive phenomenology, access to contents, and inner speech. J. Conscious. Stud. 21, 74–99.

[B42] Josephson J. Josephson S. (1994). Abductive Inference. Cambridge, UK: Cambridge University Press.

[B43] Kane R. (2005). A Contemporary Introduction to Free Will. Oxford, UK: Oxford University Press.

[B44] Kassirer J. Gorry G. (1978). Clinical problem solving: a behavioral analysis. Ann. Intern. Med. 89, 245–255. 10.7326/0003-4819-89-2-245 677593

[B45] Katz G. Huang D. Gentili R. Reggia J. (2016). “Imitation learning as cause-effect reasoning,” in 9th Conf. on Artificial General Intelligence (New York, NY: Springer Intl. Publishing).

[B46] Katz G. Huang G. Hauge T. Gentili R. Reggia J. (2017a). “A novel parsimonious cause-effect reasoning algorithm for robot imitation and plan recognition,” in IEEE Trans. on Cognitive and Developmental Systems.

[B47] Katz G. Huang D. Gentili R. Reggia J. (2017b). “An empirical characterization of parsimonious intention inference for cognitive-level imitation learning,” in Proc. 19th Intl. Conf. on AI (Los Vegas).

[B48] Katz G. Dullnig D. Davis G. Gentili R. Reggia J. (2017c). “Autonomous causally-driven explanation of actions,” in International Symposium on Artificial Intelligence (Los Vegas).

[B49] Kautz H. Allen J. (1986). “Generalized plan recognition,” in Procs. 1986 of the American Association for Artificial Intelligence, AAAI, 32–37.

[B50] Kitamura T. Tahara T. Asami K. (2000). How can a robot have consciousness? Adv. Robot. 14, 263–275. 10.1163/156855300741573

[B51] Kuipers B. (2008). Drinking from the Firehose of experience. Artif. Intell. Med. 44, 155–170. 10.1016/j.artmed.2008.07.010 18774281

[B52] Levine J. (1983). Materialism and qualia: the explanatory gap. Pac. Philos. Q. 64, 354–361. 10.1111/j.1468-0114.1983.tb00207.x

[B53] Lewis S. J. Slabosz A. Robbins T. W. Barker R. A. Owen A. M. (2005). Dopaminergic basis for deficits in working memory but not attentional set-shifting in Parkinson’s disease. Neuropsychologia 43, 823–832. 10.1016/j.neuropsychologia.2004.10.001 15716155

[B54] Massimini M. Ferrarelli F. Huber R. Esser S. K. Singh H. Tononi G. (2005). Breakdown of cortical effective connectivity during sleep. Science 309, 2228–2232. 10.1126/science.1117256 16195466

[B55] Meltzoff M. (1995). Understanding the intentions of others. Dev. Psychol. 31, 838–850. 10.1037/0012-1649.31.5.838 25147406 PMC4137788

[B56] Murphy N. Ellis G. O’Connor T. (eds) (2009). Downward Causation and the Neurobiology of Free Will. New York, NY: Springer.

[B57] Nagataki S. Hirose S. (2007). Phenomenology and the third generation of cognitive science: towards a cognitive phenomenology of the body. Hum. Stud. 30, 219–232. 10.1007/s10746-007-9060-y

[B58] Pasquali A. Timmermans B. Cleeremans A. (2010). Know thyself: metacognitive networks and measures of consciousness. Cognition 117, 182–190. 10.1016/j.cognition.2010.08.010 20825936

[B59] Peng Y. Reggia J. (1990). Abductive Inference Models for Diagnostic Problem-Solving. New York: Springer-Verlag.

[B60] Peters S. K. Dunlop K. Downar J. (2016). Cortico-striatal-thalamic loop circuits of the salience network: a central pathway in psychiatric disease and treatment. Front. Syst. Neurosci. 10:104. 10.3389/fnsys.2016.00104 28082874 PMC5187454

[B61] Prinz J. (2011). The sensory basis of cognitive phenomenology. Cogn. Phenomenol. 174 10.1093/acprof:oso/9780199579938.003.0008

[B62] Reggia J. (2013). The rise of machine consciousness. Neural Netw. 44, 112–131. 10.1016/j.neunet.2013.03.011 23597599

[B63] Reggia J. Huang D. Katz G. (2015). Beliefs concerning the nature of consciousness. J. Conscious. Stud. 22, 146–171.

[B64] Reggia J. Katz G. Huang D. (2016). What are the computational correlates of consciousness? Proc. 2016 Annual International Conference on Biol. Inspir. Cogn. Archit. New York, NY.

[B65] Reggia J. Monner D. Sylvester J. (2014). The computational explanatory gap. J. Conscious. Stud. 21, 153–178.

[B66] Samsonovich A. Nadel L. (2005). Fundamental principles and mechanisms of the conscious self. Cortex 41, 669–689. 10.1016/S0010-9452(08)70284-3 16209330

[B67] Schneider W. Chein J. M. (2003). Controlled and automatic processing: behavior, theory, and biological mechanisms. Cogn. Sci. 27, 525–559. 10.1207/s15516709cog2703_8

[B68] Schroll H. Hamker F. H. (2013). Computational models of basal-ganglia pathway functions: focus on functional neuroanatomy. Front. Syst. Neurosci. 7:122. 10.3389/fnsys.2013.00122 24416002 PMC3874581

[B69] Searle J. (2004). Mind. Oxford, UK: Oxford University Press.

[B70] Spreng R. Mar R. Kim A. (2008). The common neural basis of autobiographical memory, prospection, navigation, theory of mind, and the default mode. J. Cogn. Neurosci. 21, 489–510. 10.1162/jocn.2008.21029 18510452

[B71] Sun R. (2002). Duality of the Mind. Hillsdale, NJ: Erlbaum.

[B72] Sylvester J. Reggia J. (2016). Engineering neural systems for high-level problem solving. Neural Netw. 79, 37–52. 10.1016/j.neunet.2016.03.006 27101230

[B73] Sylvester J. Reggia J. Weems S. Bunting M. (2013). Controlling working memory with learned instructions. Neural Netw. 41, 23–38. 10.1016/j.neunet.2013.01.010 23465563

[B74] Tagliazucchi E. Chialvo D. R. Siniatchkin M. Amico E. Brichant J. F. Bonhomme V. (2016). Large-scale signatures of unconsciousness are consistent with a departure from critical dynamics. J. R. Soc. Interface 13, 1–12. 10.1098/rsif.2015.1027 26819336 PMC4759808

[B75] Takeno J. (2013). Creation of a Conscious Robot. Singapore: Pan Stanford.

[B76] Tani J. (2017). Exploring Robotic Minds: Actions, Symbols, and Consciousness as Self-Organizing Dynamic Phenomena. Oxford, UK: Oxford University Press.

[B77] Taylor J. (2007). CODAM: a neural network model of consciousness. Neural Netw. 20, 983–992. 10.1016/j.neunet.2007.09.005 17935944

[B78] Tommasi G. Fiorio M. Yelnik J. Krack P. Sala F. Schmitt E. (2015). Disentangling the role of cortico-basal ganglia loops in top-down and bottom-up visual attention: an investigation of attention deficits in Parkinson disease. J. Cogn. Neurosci. 27, 1215–1237. 10.1162/jocn_a_00770 25514652

[B79] Tononi G. (2004). An information integration theory of consciousness. BMC Neurosci. 5:42 10.1186/1471-2202-5-42 15522121 PMC543470

[B80] Vinner E. Israelashvili M. Bar-Gad I. (2017). Prolonged striatal disinhibition as a chronic animal model of tic disorders. J. Neurosci. Methods 292, 20–29. 10.1016/j.jneumeth.2017.03.003 28268105

[B81] Vreeling F. W. Verhey F. R. Houx P. J. Jolles J. (1993). Primitive reflexes in Parkinson’s disease. J. Neurol. Neurosurg. Psychiatry 56, 1323–1326. 10.1136/jnnp.56.12.1323 8270937 PMC1015384

[B82] Walton D. (2005). Abductive Reasoning. Tuscaloosa: University of Alabama Press.

[B83] Wang M. He B. (2014). A cross-modal investigation of the neural substrates for ongoing cognition. Front. Psychol. 5:945. 10.3389/fpsyg.2014.00945 25206347 PMC4143722

[B84] Wiecki T. Frank M. J. (2013). A computational model of inhibitory control in frontal cortex and basal ganglia. Psychol. Rev. 120, 329–355. 10.1037/a0031542 23586447

[B85] Wurtz R. H. Hikosaka O. (1986). Role of the basal ganglia in the initiation of saccadic eye movements. Prog. Brain Res. 64, 175–190. 10.1016/S0079-6123(08)63412-3 3523602

